# Neutrophil Percentage-to-Albumin Ratio as a Prognostic Marker for Mortality in Ischemic Stroke Patients

**DOI:** 10.7150/ijms.108493

**Published:** 2025-05-28

**Authors:** Yue-xin Lu, Bao-jie Mao, Ming Wang, Shu Wan

**Affiliations:** 1 Brain Center, Affiliated Zhejiang Hospital, Zhejiang University School of Medicine, Hangzhou, China; 2 The Second School of Clinical Medicine, Zhejiang Chinese Medical University, Hangzhou, China; 3 Zhejiang Province Engineering Research Center for Precision Medicine in Cerebrovascular Diseases, Hangzhou, China

**Keywords:** Neutrophil Percentage-to-Albumin Ratio, Ischemic Stroke, Mortality, Propensity score matching, MIMIC-IV

## Abstract

**Background:** The neutrophil percentage-to-albumin ratio (NPAR) is an emerging inflammatory biomarker that has demonstrated a significant association with poor outcomes in patients with cardiovascular diseases. However, the existing evidence regarding its prognostic value in ischemic stroke (IS) patients remains limited. Our study aimed to investigate the potential of the NPAR as a prognostic indicator for all-cause mortality in patients with IS.

**Methods:** This study screened IS patients from the Medical Information Mart for Intensive Care (MIMIC-IV) database and categorized them into two groups based on NPAR values, employing propensity score matching to mitigate confounding factors. The primary outcome assessed was 90‒day mortality, and the secondary outcomes included in-hospital mortality, ICU mortality, and mortality at 30‒day and 1‒year after admission. Cox proportional hazards regression analysis and restricted cubic splines were used to explore the relationship between the NPAR and all-cause mortality in critically ill IS patients, whereas Kaplan‒Meier analysis was used to estimate survival curves. Subgroup analysis and interaction tests were performed to evaluate the robustness of the results. Receiver operating characteristic curves were computed to assess the diagnostic value of the NPAR in predicting outcomes.

**Results:** A total of 706 patients (53.3% male) were included in the study, with in-hospital and ICU mortality rates of 18.2% and 12.6%, respectively. The mortality rates at 30‒day, 90‒day, and 1‒year were 19.2%, 29.7%, and 37.8%, respectively. Restricted cubic splines indicated a nonlinear increase in all-cause mortality as the NPAR increased. Multivariate Cox regression analysis revealed a significant association between a high NPAR and all-cause mortality at 90‒day (hazard ratio [HR]: 1.99; 95% confidence interval [95% CI]: 1.44-2.76, p < 0.001), 30‒day (HR: 2.09; 95% CI: 1.39-3.13, p < 0.001), and 1‒year (HR: 1.77; 95% CI: 1.32-2.37, p < 0.001). The subgroup analysis indicates that a significant interaction was observed between hypertension and mortality risk in IS patients (p for interaction = 0.012), suggesting that hypertension may be an important predictor of poor prognosis in these patients. Receiver operating characteristic curves demonstrated that the NPAR provides a modestly greater ability to predict the risk of death in patients with IS compared to the individual indices of neutrophil percentage and albumin levels, although the specificity (0.567) and sensitivity (0.684) of NPAR were not outstanding overall.

**Conclusion:** Our study revealed an independent association between a high NPAR and increased all-cause mortality at 30‒day, 90‒day, and 1‒year and during hospitalization in patients with IS, reinforcing its status as an independent determinant of mortality risk.

## Introduction

Ischemic stroke (IS) remains a formidable global health challenge, accounting for approximately 43.5%-76.1% of acute stroke admissions 1. It is a leading cause of disability and mortality worldwide 2-4. Following a stroke, the massive release of catecholamines and cytokines triggers a systemic inflammatory response. Reperfusion therapies expose infarcted regions to peripheral immune cells, thereby exacerbating immune activation and inflammatory damage. Neutrophils, among the first inflammatory cells recruited to the brain 5, infiltrate the brain parenchyma within 12 hours of an IS event, and their levels peak between 2 and 7 days 5, 6. During the initial stages of a stroke, neutrophils migrate to ischemic brain areas and become activated, promoting the release of reactive oxygen species, chemokines, and other mediators and further promoting inflammation. Albumin plays multiple roles, including osmoregulation and antioxidation, and is crucial in the scavenging of reactive oxygen species 7, 8. Albumin constitutes more than half of the total protein in the serum and can mitigate inflammation by inhibiting neutrophil migration 9. Despite advancements in intravascular thrombectomy and intravenous thrombolysis, which facilitate rapid reperfusion, the risk of adverse outcomes in IS patients remains significant 10. Accurately predicting the long-term effects of IS patients remains challenging. Early identification of patients at greater risk of poor outcomes is vital for optimizing treatment and rehabilitation strategies, potentially enhancing the quality of life of stroke survivors.

The critical role of neutrophils in the pathogenesis of IS has been well established 11, 12, and albumin is essential in various physiological processes, serving as an important marker of nutritional status 13. The neutrophil percentage-to-albumin ratio (NPAR) has emerged as a novel biomarker for assessing inflammation and nutritional status. It has been found to be associated with adverse clinical outcomes in cardiovascular diseases, such as chronic coronary artery disease, atrial fibrillation, heart failure, acute myocardial infarction, and cardiogenic shock 14-18.

The value of NPAR in predicting the recurrence of first episode IS and the occurrence of stroke-associated infections has been explored 19, 20. However, the prognostic value of the NPAR in predicting outcomes for patients with IS patients remains uncertain. This study utilized the Medical Information Mart for Intensive Care (MIMIC-IV) database (version 3.0) to explore the correlation between NPAR at admission and mortality in patients with IS.

## Methods

### Data Source and Data Privacy

This study obtained health-related data from the publicly accessible MIMIC-IV database (version 3.0). The MIMIC-IV has received approval from the Massachusetts Institute of Technology (MIT, Cambridge, MA, USA) and the Institutional Review Boards of Beth Israel Deaconess Medical Center (BIDMC, Boston, MA, USA). providing a wealth of anonymized clinical data relevant to the care of ICU patients. The MIMIC-IV serves as a valuable tool for investigating predictive modeling, outcomes in critical care, and other research areas.

To ensure adherence to ethical standards and protect patient privacy, the data utilized in this study were deidentified. Author Yue-xin Lu completed the web-based courses on "Conflict of Interest" and "Data or Specimens Only Research" at an institution affiliated with MIT (record ID: 12901560), thereby obtaining authorization to access and extract data from the MIMIC-IV database.

### Study Population

**Figure 1:**
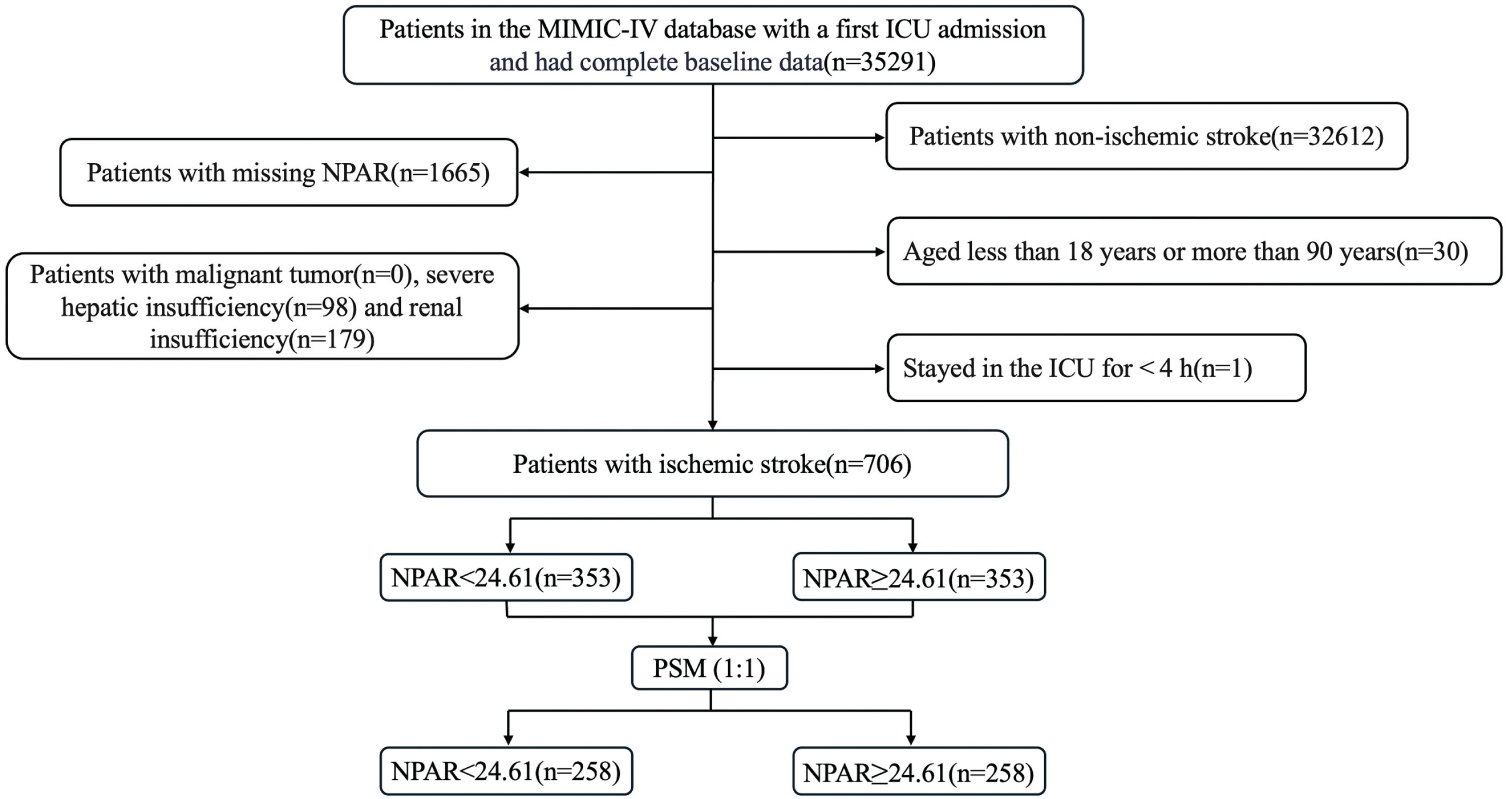
Flow chart of enrolled patients.

This retrospective study included patients diagnosed with IS based on the International Classification of Diseases, Ninth and Tenth Revisions. The exclusion criteria were as follows: (1) patients admitted for the first time at age under 18 years or over 90 years; (2) patients who had multiple ICU admissions due to IS, and only data from the initial admission were extracted; (3) patients with an ICU stay of less than 4 hours, to exclude the confounding factors from patients with very short-term (e.g., rapid death, rapid transfer out) ICU stays; (4) patients with severe comorbidities such as liver or renal failure or cancer; and (5) patients whose key data such as NPAR were missing. Ultimately, 706 patients were included in this study and divided into two groups based on the median NPAR. The inclusion flow chart is shown in Fig. 1.

### Data Collection and Management of Missing Values

Information was extracted from a structured query language (SQL) via PostgreSQL version 16.4 and Navicat Premium version 17. Extracted variables were categorized into five main groups: (1) demographic data, including age, sex, height, and weight; (2) severity scores at admission, including the Acute Physiology Score III, Oxford Acute Severity of Illness Score, Glasgow Coma Scale (GCS), and the Sequential Organ Failure Assessment (SOFA) score; (3) comorbidities, including arrhythmias, hypertension, diabetes, heart failure, coronary heart disease, chronic obstructive pulmonary disease, and cerebral hemorrhage; (4) laboratory markers, such as albumin, neutrophil percentage, red blood cells, hemoglobin, platelets, white blood cells, lymphocyte percentage, creatinine, urea nitrogen, glucose, sodium, potassium, chloride, lactate, and bicarbonate; and (5) medication history, including anticoagulants, antiplatelet agents, and lipid-lowering drugs, and treatments received postadmission, such as mechanical thrombectomy or thrombolysis. All laboratory markers and severity scores were extracted exclusively from data collected within the initial 24 hours after ICU admission. Each subject's observation spanned from admission until the occurrence of a death event.

The primary outcome measure in our study was 90-day all-cause mortality, with secondary outcomes including 30-day and 1-year mortality rates as well as ICU mortality and in-hospital mortality rates. Crucially, the definition of death was time-bound to the postadmission period, not post-ICU entry. The NPAR was calculated using the following formula: NPAR = neutrophil percentage (%) * 100/albumin (g/dl).

To mitigate potential bias arising from missing data, variables with a proportion of missing values exceeding 20% were excluded from the study. The "mice" package in R software was utilized for multiple imputation of variables with less than 20% missing data.

### Statistical Analysis

Continuous variables are presented as medians with interquartile ranges and were analyzed using the Mann‒Whitney U test. Categorical variables are represented as counts with proportions and were compared via the chi-square test.

The process of patient selection may introduce selection bias and potential confounders. Therefore, to minimize the effects of bias and confounding factors, we performed a propensity score matching (PSM) analysis using variables such as age, weight, height, GCS score, SOFA score, APS III score, hypertension, diabetes, arrhythmias, heart failure, coronary heart disease, chronic obstructive pulmonary disease, cerebral hemorrhage, mechanical thrombectomy, thrombolysis, Antiplatelet, Anticoagulation, and Lipid-lowering drug. Logistic regression models were created to calculate propensity scores. The PSM analysis was performed via a 1:1 nearest neighbor matching algorithm with a caliper set at 0.1. To assess the level of balance between the groups, we calculated the standardized mean differences (SMDs) before and after matching. An SMD less than 0.10 indicates a well-balanced distribution of characteristics between the matched groups.

To observe the overall impact trend of NPAR values on the mortality of IS patients, we used restricted cubic splines (RCS) to analyze the potential nonlinear relationship between the NPAR and all-cause mortality. We utilized Kaplan‒Meier (KM) curves to visualize endpoint incidence rates in survival analysis. Univariate and multivariate analyses were performed using Cox proportional hazards models to control for confounding factors, providing more accurate predictions of patient mortality following an IS. Variables related to clinical outcomes and prognosis were included in the multivariate models: Model 1 was unadjusted; Model 2 was adjusted for age, sex, and race; and Model 3 was adjusted for age, sex, arrhythmias, heart failure, hypertension, diabetes, mechanical thrombectomy, thrombolysis, red blood cells, white blood cells, platelets, and SOFA score. The results are reported as hazard ratios (HRs) with 95% confidence intervals (CIs). Subgroup analyses were also conducted to explore the potential impact of different subgroups (including sex, age, comorbidities, and various physiological scores) on outcomes. The predictive performance of NPAR for prognosis of IS patients was assessed using the area under the receiver operating characteristic (ROC) curve, with the optimal NPAR cutoff value determined using the Youden index. A bilateral P-value < 0.05 was considered statistically significant.

All the statistical analyses were performed via STATA 14.0, R software (version 4.4.2) and SPSS 29.0 (IBM SPSS Statistics, Armonk, NY, USA).

## Results

### Baseline Characteristics Before and After PSM

In this study, a total of 706 critically ill patients with IS were included. The baseline characteristics of patients are shown in Table 1. The median NPAR for all included participants was 24.61 (IQR: 8.92). The median age of the patients was 68 years, with 376 (53.3%) being male and more than half (55.0%) of the patients were White.

Before PSM, individuals were divided into two groups based on median NPAR. Compared with the group with a lower NPAR, the group with a higher NPAR had lower GCS scores; red blood cell, hemoglobin, and bicarbonate levels; and a lower proportion of patients on lipid-lowering medications. The higher NPAR group also presented an increased incidence of arrhythmias, higher white blood cell, creatinine, and urea levels.

**Figure 2:**
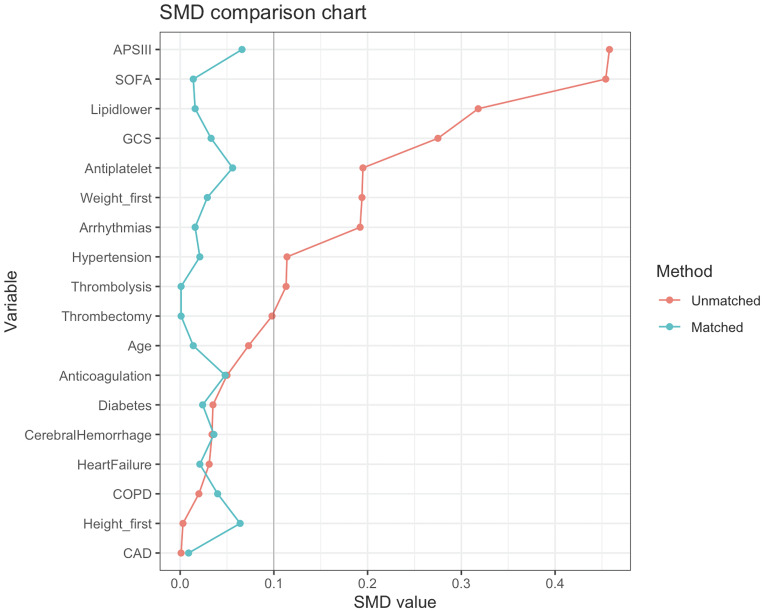
SMDs before and after PSM.

To minimize baseline characteristic differences between the low NPAR and high NPAR patient groups, a 1:1 PSM analysis was conducted, resulting in 258 matched pairs. The baseline characteristics of patients after PSM are shown in Table 2. The two cohorts demonstrated balanced demographics, comorbidities, disease severity scores, and treatments. The effectiveness of the matching process was assessed by calculating the SMD before and after PSM, as depicted in Fig. 2.

### NPAR and All-Cause Mortality

The clinical outcomes before PSM are shown in Table 3. Higher NPAR group had a longer hospital stays (20 vs. 16 days, P < 0.001) and ICU stays (9 vs. 6 days, P < 0.001); as well as higher rates of in-hospital mortality (23.8 vs. 12.5%, P < 0.001) and ICU mortality (15.0 vs. 8.5%, P = 0.007). Mortality rates at 90‒day (40.5 vs. 19.5%, P < 0.001), 30‒day (26.3 vs. 12.7%, P < 0.001), and 1‒year (48.7 vs. 26.3%, P < 0.001) were also significantly higher in the group with a higher NPAR.

The clinical outcomes after PSM are shown in Table 4. Significant differences were noted between the two cohorts in all-cause mortality at 90‒day (36.8% vs. 22.5%, p < 0.001), 30‒day (23.6% vs. 14.7%, p = 0.010), 1‒year (45.0% vs. 30.6%, p < 0.001). The duration of hospital stays (p = 0.001), and ICU stay (p = 0.027) also show significant differences. No significant differences were observed in ICU all-cause mortality rates (14.7% vs. 10.5%, p = 0.144) or in-hospital mortality rates (20.9% vs. 15.5%, p = 0.110).

### RCS and KM Curves Analyze

**Figure 3:**
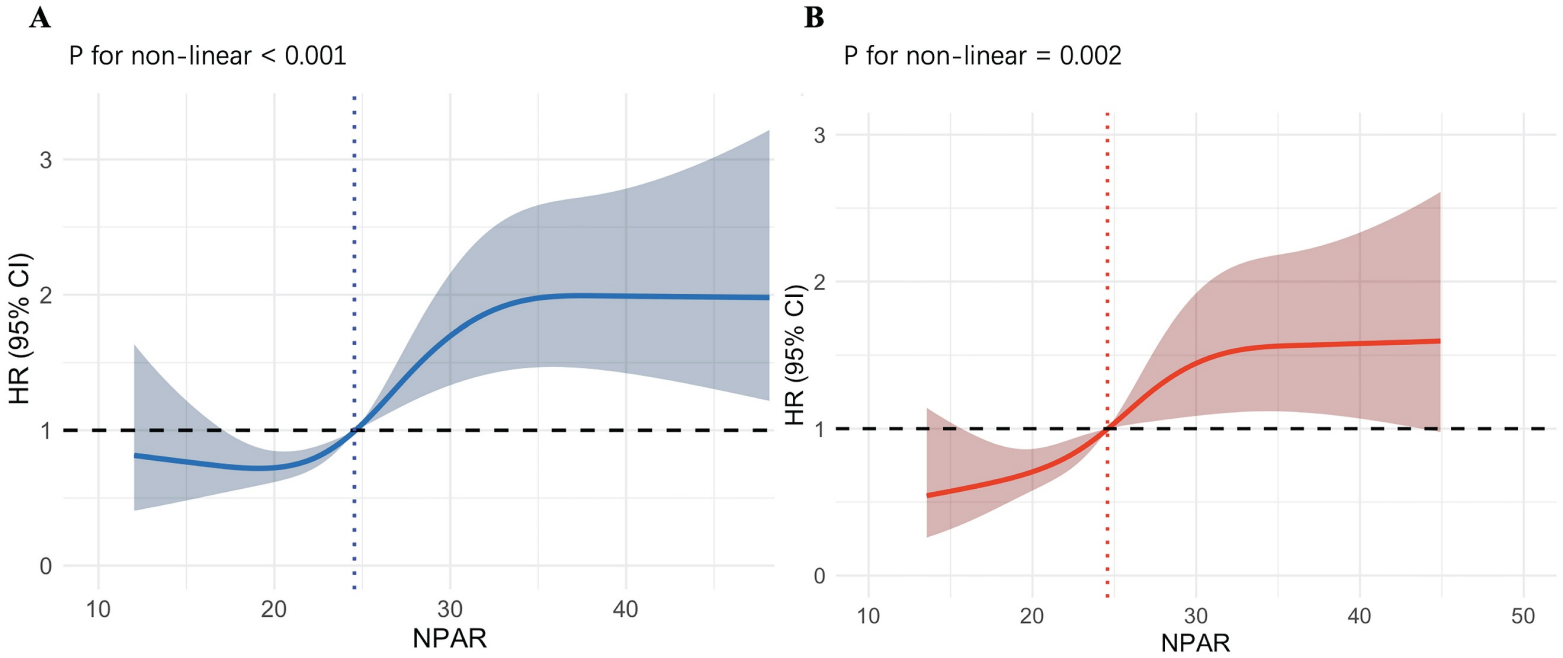
Underlying nonlinear correlations between NPAR and 90-day all-cause mortality of IS patients before and after PSM.

As shown in Fig. 3A, we employed RCS to explore the nonlinear relationship between the NPAR and all-cause mortality in IS patients. The X-axis represents the NPAR value, and the Y-axis represents the HR (95% CI) for IS patients at 90‒day. The shaded area represents the 95% confidence interval. We observed that increases in NPAR values are nonlinearly correlated with 90-day mortality rates (p for nonlinearity < 0.001). When NPAR is less than 24.55, it is associated with a lower risk of death. When NPAR exceeds 24.55, the HR is greater than 1, and particularly when NPAR is greater than 30, the HR remains at approximately 2, indicating an association with a higher risk of death. As shown in Fig. 3B, the nonlinear association between increased NPAR values and 90-day mortality in IS patients persisted after performing PSM (p for nonlinearity = 0.002). Using an NPAR value of 24.58 as the cutoff, the risk of death is lower when the value is below it, and higher when the value is above it. Overall, the entire curve shows a trend of increasing death risk as the NPAR value rises. The RCS plots of the NPAR values and mortality rates at 30‒day, 1‒year, during ICU stays, and during hospitalization in patients with IS before and after PSM are provided in the supplement.

**Figure 4:**
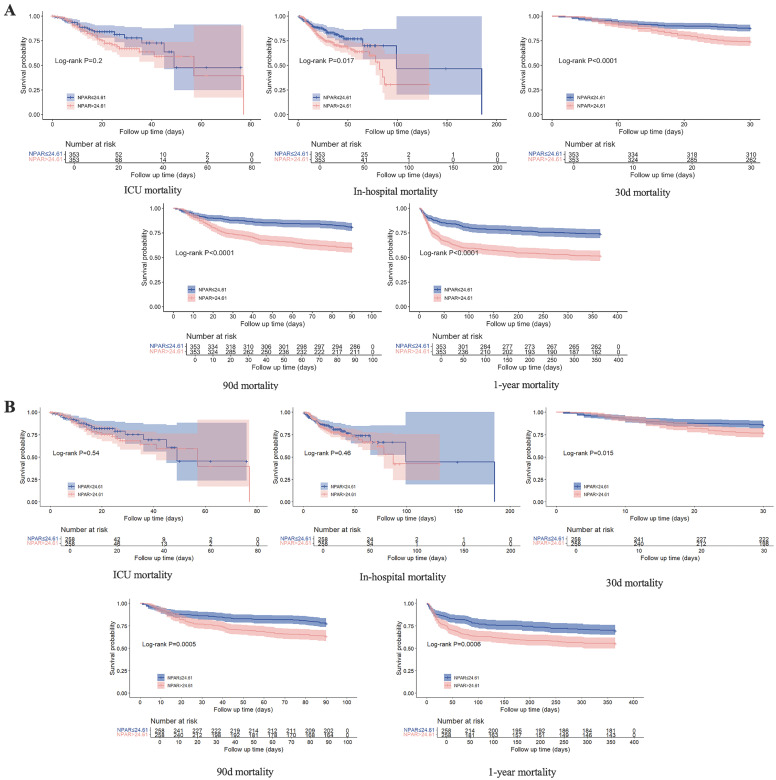
KM curve plots of 30-day, 90-day, and 1-year survival rates and in-hospital and ICU stay survival rates of IS patients before and after PSM.

The KM curve (Fig. 4A) revealed higher all-cause mortality at 90‒day, 30‒day, and 1‒year, as well as in-hospital mortality for patients with an NPAR ≥ 24.61 than for those with an NPAR < 24.61, whereas no significant difference in ICU mortality was observed between the two groups. After adjusting for confounding factors via PSM, the KM curve (Fig. 4B) revealed that IS patients with an NPAR ≥ 24.61 had significantly higher rates of 90-day, 30-day, and 1-year mortality (all P < 0.05), but there was no notable disparity in either ICU mortality or in-hospital mortality.

### Univariate and Multivariate Cox Regression Analyses

To investigate the potential associations between the NPAR and mortality outcomes in IS patients, we conducted univariate and multivariate Cox regression models. The detailed data are presented in Table 5. In the unadjusted initial model, a higher NPAR (≥ 24.61) was significantly correlated with an elevated risk of mortality at various time intervals, including 90‒day (HR = 2.36, 95% CI: 1.77-3.14, p < 0.001), 30‒day (HR = 2.20, 95% CI: 1.54-3.14, p < 0.001), and 1‒year (HR = 2.19, 95% CI: 1.70-2.82, p < 0.001), as well as an increased in-hospital mortality rate (HR = 1.56, 95% CI: 1.08-2.26, p = 0.018). After adjusting for potential confounders, including age, sex, and race, in multivariate Model 1, the patient group with an NPAR ≥ 24.61 continued to have significantly higher mortality risks at the aforementioned time points. Another multivariate model (Model 2) further established that an elevated NPAR was independently associated with increased risks of mortality at 90‒day, 30‒day and 1‒year, although it no longer showed a significantly elevated risk of in-hospital mortality.

Furthermore, multivariate Cox regression analyses post-PSM indicated that an NPAR ≥ 24.61 remained a predictive marker for increased risk of mortality at 90‒day (HR = 1.99, 95% CI: 1.44-2.76, p < 0.001), 30‒day (HR = 2.09, 95% CI: 1.39-3.13, p < 0.001), and 1‒year (HR = 1.77, 95% CI: 1.32-2.37, p < 0.001). These results suggest that a higher NPAR is strongly associated with increased short- and long-term mortality risk in IS patients.

### Subgroup Analysis

**Figure 5:**
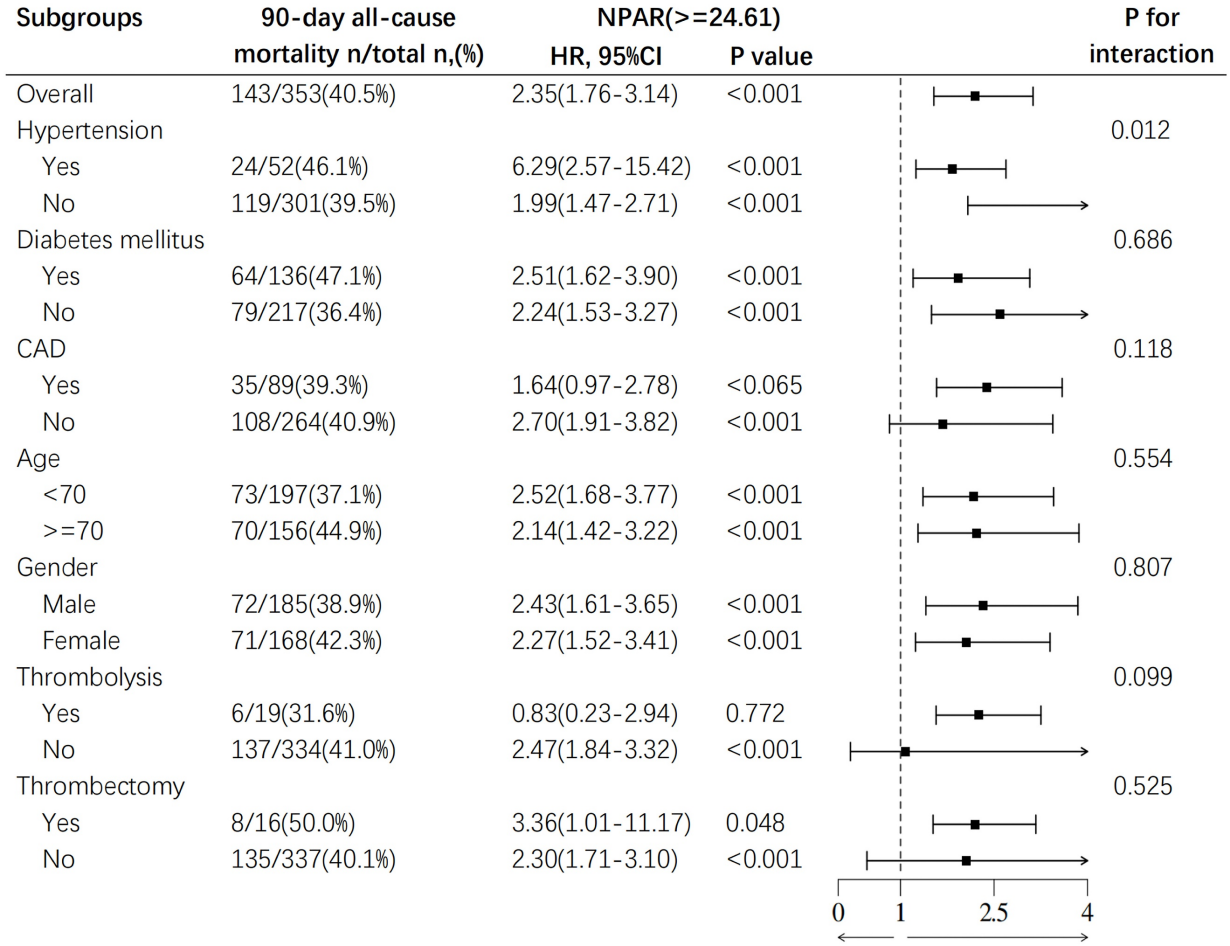
Subgroup analysis for the effect of NPAR on 90-day all-cause mortality in patients with IS.

To further evaluate the relationship between NPAR and mortality, we conducted subgroup analysis to assess the associations between age (< 70 years and ≥ 70 years), sex, hypertension, diabetes, coronary artery disease, pharmacological thrombolysis, mechanical thrombectomy, and all-cause mortality at 90‒day in IS patients. The results revealed a correlation between a higher NPAR and increased 90‒day all-cause mortality as illustrated in Fig. 5. A significant interaction was observed between hypertension and the mortality risk in IS patients (p for interaction = 0.012). The mortality risk for IS patients with hypertension (HR 6.29, 95% CI: 2.57-15.42, p < 0.001) was significantly higher than that for patients without hypertension (HR 1.99, 95% CI: 1.47-2.71, p < 0.001), suggesting that hypertension may be an important predictor of poor prognosis in IS patients. No other subgroup factors significantly affected the relationship between NPAR and 90-day all-cause mortality. Subgroup analyses for 30-day, 1‒year, and ICU mortality and in-hospital mortality are detailed in the supplement.

### ROC Curve Analysis

**Figure 6:**
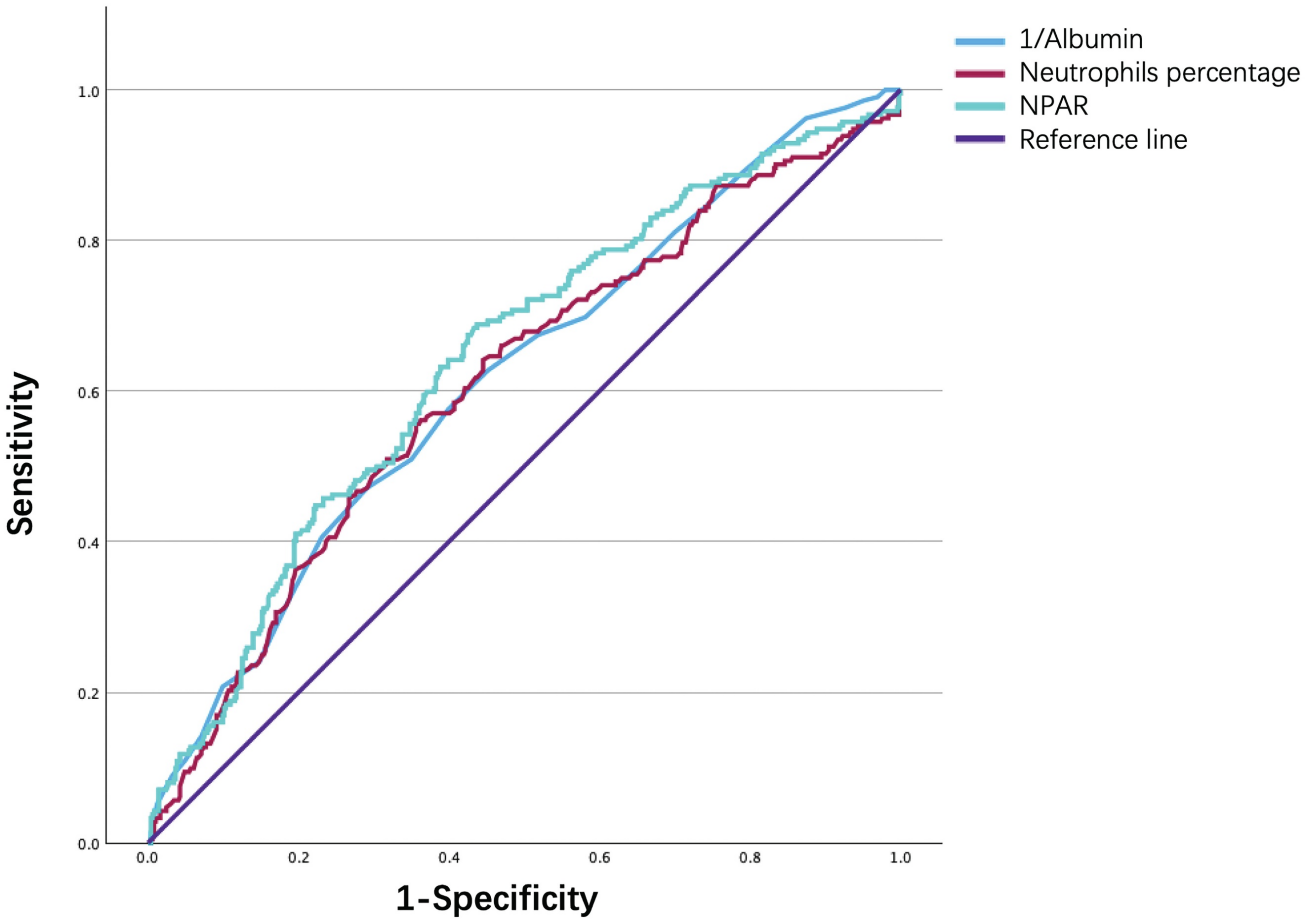
ROC curve analysis of NPAR in predicting 90-day mortality of ischemic stroke patients.

Finally, the ROC curve in Fig. 6. illustrates the ability of the NPAR, neutrophil percentage, and albumin level to predict 90‒day mortality in IS patients. As shown in Table 6, although all three biomarkers exhibited limited discriminative power, the NPAR demonstrated a modestly higher predictive value, with an area under the curve of 0.641, sensitivity of 0.684, and specificity of 0.567, compared to the neutrophil percentage [0.610 (0.564-0.656)] and the serum ALB concentration [0.616 (0.571-0.661)], with a cutoff value of 24.48.

## Discussion

In this study, we investigated the associations between the NPAR values and clinical outcomes in patients with IS during hospitalization, during the ICU stay, and at 30, 90, and 365 days postadmission. The results indicate a significant correlation between elevated NPAR values and increased all-cause mortality risk at 30 days, 90 days, and 1 year. Additionally, there was a correlation with prolonged hospital stays and ICU stays. Even after adjusting for confounding factors, the NPAR remained an independent predictor of all-cause mortality in IS patients.

In our study, we found that higher NPAR values are positively correlated with the risk of death in patients with IS. The study by Xu et al. found that after adjusting for confounding factors, NPAR was positively correlated with stroke (OR: 1.09, 95% CI: 1.05 ~ 1.12) 21, suggesting that NPAR may serve as a potential inflammatory marker reflecting the occurrence of stroke. Lv et al. reported in their study that elevated NPAR is associated with poor outcomes at 3 months in patients with spontaneous intracerebral hemorrhage 22 (adjusted odds ratio: 1.72; 95% confidence interval, 1.03-2.90; P=0.040), which is consistent with our findings.

Considering the potential impact of confounding factors on the outcomes, we performed PSM and adjusted for some potential confounders in the Cox regression analysis. Age and gender may alter the association between NPAR and clinical outcomes by affecting individual immune function. As age increases, the inflammatory response may become more pronounced 23. Additionally, Gender is a biological variable that affects the functions of the immune system which may play a different role in certain inflammatory responses 24. Comorbidities such as hypertension, diabetes, and coronary artery disease have been established as most common risk factors in stroke patients. These conditions may exacerbate the pathological process of stroke by triggering chronic inflammation and endothelial injury. During treatment, a series of acute inflammatory responses may occur, especially during the reperfusion phase 25. Mechanical thrombectomy and thrombolysis may affect NPAR levels by altering the vascular environment or inducing further leukocyte infiltration, thereby influencing its association with prognosis. The SOFA score, a clinical scoring tool used in intensive care, reflects the extent of multi-organ dysfunction. Antiplatelet and anticoagulant medications may indirectly affect the predictive value of NPAR by altering leukocyte responses in the blood or modifying levels of inflammatory mediators 26, 27. Lipid-lowering drugs (such as statins) can impact stroke outcomes by reducing inflammation and improving endothelial function 28.

Despite early diagnosis and timely intervention, IS continues to represent a major cause of disability and death worldwide. Inflammation plays a crucial role in the development of IS, contributing to initial brain damage 29. NPAR, as a marker integrating the neutrophil percentage-to-albumin ratio, can reflect the severity of systemic inflammatory response. In IS, the inflammatory response is not limited to brain tissue damage but may also be activated through systemic inflammation, including leukocytosis and cytokine release in peripheral blood. Patients with IS typically experience varying degrees of blood-brain barrier disruption and reperfusion injury during the acute phase 30, which makes the inflammatory response more prominent and persistent. During reperfusion injury, leukocytes, especially neutrophils, rapidly infiltrate the damaged brain tissue, which not only exacerbates the local inflammatory response but also may lead to hemorrhagic transformation. Although the infiltration of leukocyte subtypes into the brain postischemia may vary with the timing of reperfusion, most studies indicate that the detrimental effects of neutrophils are more pronounced during reperfusion 31, 32. The level of albumin, which constitutes more than half of the total serum protein content, reflects an individual's nutritional status and inflammation level. Low albumin levels often indicate malnutrition, which can be due to dietary deficiencies, chronic diseases, or inflammation. A decrease in albumin may lead to increased blood viscosity, which can affect cerebral perfusion and increase the risk of ischemic brain injury. During the acute phase of IS, albumin is crucial in mitigating the adverse effects of inflammation associated with neuronal injury. However, this inflammatory state can lead to reduced serum ALB levels 33. Up to 45.5% of patients exhibit hypoalbuminemia (albumin levels < 35 g/L) post-AIS 34, and those with lower albumin levels have a higher recurrence rate and mortality from stroke 35. Previous research has shown that reduced serum ALB levels are independently associated with poor outcomes in patients with anterior circulation acute large vessel occlusive strokes treated with endovascular thrombectomy 36. Albumin also displays neuroprotective effects during IS reperfusion by inhibiting thrombosis and leukocyte adhesion in the microvascular system 37.

The NPAR, a novel marker of systemic inflammation that integrates the percentage of neutrophils with the serum ALB concentration, reflects adverse outcomes in IS patients more effectively than single markers do. Furthermore, the NPAR has greater predictive potential than other composite indices, such as the C-reactive protein-to-albumin ratio and neutrophil-to-lymphocyte ratio 38. Although the NIHSS score reflects neurological damage in the acute phase of stroke, it does not fully capture the extent of the systemic inflammatory response. Therefore, we recommend incorporating NPAR into existing prognostic scores for IS risk stratification, with regular monitoring of patients' neutrophil percentage and serum albumin levels and calculating the NPAR value. This not only helps in the early detection of abnormal inflammation levels in patients but also provides a basis for treatment decisions. In patients with elevated NPAR, regulating the inflammatory response may become a key intervention strategy. For example, using anti-inflammatory treatments or adopting immunosuppressive strategies may help reduce neutrophil-induced damage to brain tissue, thereby lowering the risk of stroke recurrence and the occurrence of other long-term complications and improving patient outcomes.

The major strength of this study lies in our analysis of the extensive public MIMIC-IV database, which confirmed that a high NPAR was a significant predictor of increased mortality in critically ill patients with IS. However, there are several limitations to our study. First, as a single-center retrospective study, our findings are limited by the inherent biases associated with retrospective data, and the generalizability of the NPAR's ability to predict mortality in IS patients requires validation in future prospective, multicenter studies. Second, similar to previous studies, we only calculated initial NPAR values after the onset of IS; the dynamic changes in NPAR values during disease progression may also be significantly correlated with the prognosis of IS. Future research should include longitudinal monitoring of NPAR values during hospitalization to better understand its temporal variability and potential predictive value over time. Third, we excluded patients with missing baseline or critical data and eliminated indicators with more than 20% missing values, such as triglycerides, lipoproteins, and CRP, in our preliminary analysis to ensure the completeness and reliability of our findings, although these could be significant confounders and may lead to potential biases. Fourth, it is important to note that the study population was selected from ICU wards, which may limit the generalizability of the results to the broader population of IS patients. Fifth, the subgroup analysis may be influenced by the relatively small sample size, and larger, more robust studies are needed to confirm these findings.

## Conclusion

In summary, NPAR served as a biomarker for all-cause mortality in patients with IS; a higher NPAR was associated with an increased risk of all-cause mortality. Future research should focus on further exploring its integration into clinical decision-making algorithms to enhance its practical application in clinical settings.

## Supplementary Material

Supplementary figures and tables.

## Figures and Tables

**Table 1:** Baseline characteristics before PSM.

Variable	Total (n=706)	NPAR			P value
		<24.61 (n=353)	≥24.61 (n=353)		
**Demographics**					
Age, years	68 (18)	67 (19)	68 (17)		0.350
Men, n (%)	376 (53.3%)	191 (54.1%)	185 (52.4%)		0.651
Race, n (%)					0.907
White	388 (55.0%)	190 (53.8%)	198 (56.1%)		
Black	71 (10.1%)	35 (9.9%)	36 (10.2%)		
Asian	30 (4.2%)	15 (4.2%)	15 (4.2%)		
Others	217 (30.7%)	113 (32.0%)	104 (29.5%)		
Height, inch	66 (7)	66 (7)	66 (6)		0.382
Weight, bls	169 (61)	174 (65)	166 (60)		0.014
**Clinical severity**					
GCS	10 (8)	11 (7)	9 (9)		<0.001
SOFA	5 (5)	4 (4)	6 (6)		<0.001
OASIS	34 (6)	32 (10)	35 (13)		<0.001
APS III	42 (24)	38 (21)	47 (26)		<0.001
**Comorbidities**					
Hypertension, n (%)	119 (16.9%)	67 (19.0%)	52 (14.7%)		0.132
Diabetes mellitus, n (%)	266 (37.7%)	130 (36.8%)	136 (38.5%)		0.641
COPD, n (%)	60 (8.5%)	31 (8.8%)	29 (8.2%)		0.787
Heart failure, n (%)	24 (3.4%)	13 (3.7%)	11 (3.1%)		0.678
Arrhythmias, n (%)	281 (39.8%)	124 (35.1%)	157 (44.5%)		0.011
CAD, n (%)	178 (25.2%)	89 (25.2%)	89 (25.2%)		1.000
Cerebral hemorrhage, n (%)	88 (12.5%)	46 (13.0%)	42 (11.9%)		0.649
** Laboratory parameters**					
RBC, K/uL	3.7 (1.2)	3.9 (1.1)	3.4 (1.2)		<0.001
WBC, m/uL	11.8 (7)	10.8 (6)	13.1 (8)		<0.001
Platelet, K/uL	200 (128)	204 (65)	196 (138)		0.166
Hemoglobin, g/dl	10.8 (3.7)	11.7 (3.4)	10.1 (3.7)		<0.001
Lymphocytes, %	11.5 (11.2)	16.3 (11.6)	8.4 (7.1)		<0.001
Sodium, mEq/L	139 (6)	139 (5)	138 (6)		0.122
Glucose, mg/dl	134 (69)	130 (68)	138 (72)		0.076
Potassium, mEq/L	4.0 (0.8)	4.0 (0.8)	4.1 (0.9)		0.105
Creatinine, mg/dl	0.9 (0.6)	0.9 (0.5)	1.0 (0.8)		0.002
Lactate, mmol/L	1.6 (1.2)	1.7 (1.1)	1.6 (1.4)		0.702
Chloride, mEq/L	105 (7)	105 (7)	105 (9)		0.701
Bicarbonate, mEq/L	22 (5)	23 (4)	22 (5)		<0.001
Urea, mg/dl	18.0 (14.0)	16.0 (10.0)	20.0 (18.5)		<0.001
**Treatment**					
Thrombolysis, n (%)	30 (4.2%)	11 (3.1%)	19 (5.4%)		0.136
Thrombectomy, n (%)	40 (5.7%)	24 (6.8%)	16 (4.5%)		0.193
**Medication**					
Antiplatelet	447 (63.3%)	240 (68.0%)	207 (58.6%)		0.010
Anticoagulation	612 (86.7%)	303 (85.8%)	309 (87.5%)		0.506
Lipid-lowering	305 (43.2%)	180 (51.0%)	125 (35.4%)		<0.001
					

Abbreviations: GCS, Glasgow Coma Scale; SOFA, sequential organ failure assessment; OASIS, Oxford acute severity of illness score; APSIII, acute physiology score III; COPD, chronic pulmonary disease; CAD, coronary atherosclerotic heart disease; RBC, red blood cell; WBC, white blood cell.

**Table 2:** Baseline characteristics after PSM.

Variable	Total (n=516)	NPAR			P value
		<24.61 (n=258)	≥24.61 (n=258)		
**Demographics**					
Age, years	67.7 (18)	67.8 (18.4)	67.6 (17.6)		0.784
Men, n (%)	272 (52.7%)	136 (52.7%)	136 (52.7%)		1.000
Race, n (%)					0.875
White	288 (55.8%)	148 (57.4%)	140 (54.3%)		
Black	47 (9.1%)	23 (8.9%)	24 (9.3%)		
Asian	23 (4.5%)	12 (4.7%)	11 (4.3%)		
Others	158 (30.6%)	83 (32.2%)	75 (29.1%)		
Height, inch	66 (7)	66 (6)	66 (6)		0.376
Weight, bls	167 (58)	165 (57)	168 (59)		0.955
**Clinical severity**					
GCS	10 (6-14)	10 (8)	10 (8)		0.705
SOFA	5 (3-7)	5 (4)	5 (4)		0.862
APS III	42 (32-54)	41 (23)	42 (22)		0.559
OASIS	34 (28-39)	34 (10)	34 (11)		0.716
**Comorbidities**					
Hypertension, n (%)	88 (17.1%)	45 (17.4%)	43 (16.7%)		0.815
Diabetes mellitus, n (%)	193 (37.4%)	98 (38.0%)	95 (36.8%)		0.785
COPD, n (%)	49 (9.5%)	23 (8.9%)	26 (10.1%)		0.652
Heart failure, n (%)	19 (3.7%)	10 (3.9%)	9 (3.5%)		0.815
Arrhythmias, n (%)	204 (39.5%)	101 (39.1%)	103 (39.9%)		0.857
CAD, n (%)	133 (25.8%)	67 (26.0%)	66 (25.6%)		0.920
Cerebral hemorrhage, n (%)	63 (12.2%)	33 (12.8%)	30 (11.6%)		0.687
** Laboratory parameters**					
RBC, K/uL	3.6 (1.2)	3.8 (1.1)	3.4 (1.1)		<0.001
WBC, m/uL	11.9 (7.3)	10.7 (6.0)	13.4 (7.7)		<0.001
Platelet, K/uL	200 (130)	200 (120)	201 (141)		0.516
Hb, g/dl	10.6 (3.7)	11.5 (3.4)	10.0 (3.5)		<0.001
Lymphocytes, %	11.1 (11.0)	16.1 (13.1)	8.5 (7.2)		<0.001
Sodium, mEq/L	139 (6)	139 (5)	138 (7)		0.136
Potassium, mEq/L	4.0(0.8)	4.0 (0.7)	4.1 (0.8)		0.596
Glucose, mg/dl	136 (71)	135 (74)	137 (67)		0.921
Creatinine, mg/dl	0.9 (0.6)	0.9 (0.5)	0.9 (0.6)		0.544
Lactate, mmol/L	1.6 (1.1)	1.6 (0.9)	1.5 (1.2)		0.151
Chloride, mEq/L	105 (7)	104 (7)	105 (8)		0.745
Bicarbonate, mEq/L	22 (5)	22 (5)	22 (5)		0.239
Urea, mg/dl	17.0 (13.0)	17.0 (12.0)	19.0 (14.0)		0.048
**Treatment**					
Thrombolysis, n (%)	22 (4.3%)	11 (4.3%)	11 (4.3%)		1.000
Thrombectomy, n (%)	28 (5.4%)	14 (5.4%)	14 (5.4%)		1.000
**Medication**					
Antiplatelet	323 (62.6%)	165 (64.0%)	158 (61.2%)		0.524
Anticoagulation	454 (88.0%)	229 (88.8%)	225 (87.2%)		0.588
Lipid-lowering	214 (41.5%)	108 (41.9%)	106 (41.1%)		0.858
					

**Table 3:** Clinical outcomes before PSM.

Variable	Total (n=554)	NPAR		P value
		< 24.61 (n=258)	≥ 24.61 (n=258)	
LOS ICU, day	7 (12)	6 (11)	9 (13)	< 0.001
LOS hospital, day	18 (20.2)	16 (19)	20 (21.5)	< 0.001
In-hospital mortality, n (%)	128 (18.1%)	44 (12.5%)	84 (23.8%)	< 0.001
ICU mortality, n (%)	83 (11.8%)	30 (8.5%)	53 (15.0%)	0.007
30-day mortality, n (%)	138 (19.5%)	45 (12.7%)	93 (26.3%)	< 0.001
90-day mortality, n (%)	212 (30.0%)	69 (19.5%)	143 (40.5%)	< 0.001
1-year mortality, n (%)	265 (37.5%)	93 (26.3%)	172 (48.7%)	< 0.001

**Table 4:** Clinical outcomes after PSM.

Variable	Total (n=516)	NPAR		P value
		< 24.61 (n=258)	≥ 24.61 (n=258)	
**Clinical outcomes after PSM**				
LOS ICU, day	7 (12)	6 (11)	8 (12)	0.027
LOS hospital, day	19 (22)	17 (20)	21 (23)	0.001
In-hospital mortality, n (%)	94 (18.2%)	40 (15.5%)	54 (20.9%)	0.110
ICU mortality, n (%)	65 (12.6%)	27 (10.5%)	38 (14.7%)	0.144
30-day mortality, n (%)	99 (19.2%)	38 (14.7%)	61 (23.6%)	0.010
90-day mortality, n (%)	153 (29.7%)	58 (22.5%)	95 (36.8%)	< 0.001
1-year mortality, n (%)	195 (37.8%)	79 (30.6%)	116 (45.0%)	< 0.001

**Table 5:** Univariate and multivariate Cox regression models of the NPAR and mortality in patients with ischemic stroke before PSM.

	Unadjusted				Model 1					Model 2				
	HR, 95% CI	P value				HR, 95% CI		P value				HR, 95% CI	P value	
**Outcomes before PSM**														
**ICU mortality**														
NPAR<24.61	1 (Ref)					1 (Ref)						1 (Ref)		
NPAR≥24.61	1.35 (0.86-2.11)			0.196		1.30 (0.83-2.04)		0.257				1.38 (0.87-2.20)	0.170	
**In-hospital mortality**														
NPAR<24.61	1 (Ref)					1 (Ref)						1 (Ref)		
NPAR≥24.61	1.56 (1.08-2.26)			0.018		1.55 (1.07-2.24)		0.021				1.57 (1.07-2.30)	0.019	
**30-day mortality**														
NPAR<24.61	1 (Ref)					1 (Ref)						1 (Ref)		
NPAR≥24.61	2.20 (1.54-3.14)			<0.001		2.18 (1.53-3.12)		<0.001				2.16 (1.48-3.14)	<0.001	
**90-day mortality**														
NPAR<24.61	1 (Ref)					1 (Ref)						1 (Ref)		
NPAR≥24.61	2.36 (1.77-3.14)			<0.001		2.34 (1.75-3.12)		<0.001				2.20 (1.63-2.98)	<0.001	
**1-year mortality**														
NPAR<24.61	1 (Ref)					1 (Ref)						1 (Ref)		
NPAR≥24.61	2.19 (1.70-2.82)			<0.001		2.18 (1.69-2.80)		<0.001				1.98 (1.51-2.58)	<0.001	
**Outcomes after PSM**														
**ICU mortality**														
NPAR<24.61	1 (Ref)						1 (Ref)				1 (Ref)			
NPAR≥24.61	1.20 (0.73-1.98)		0.477				1.21 (0.73-2.01)		0.466		1.44 (0.85-2.43)		0.173	
**In-hospital mortality**														
NPAR<24.61	1 (Ref)						1 (Ref)				1 (Ref)			
NPAR≥24.61	1.21 (0.80-1.82)		0.377				1.21 (0.80-1.83)		0.372		1.38 (0.90-2.11)		0.141	
**30-day mortality**														
NPAR<24.61	1 (Ref)						1 (Ref)				1 (Ref)			
NPAR≥24.61	1.72 (1.16-2.54)		0.007				1.75 (1.18-2.59)		0.005		2.09 (1.39-3.13)		<0.001	
**90-day mortality**														
NPAR<24.61	1 (Ref)						1 (Ref)				1 (Ref)			
NPAR≥24.61	1.78 (1.29-2.45)		<0.001				1.80 (1.31-2.48)		<0.001		1.99 (1.44-2.76)		<0.001	
**1-year mortality**														
NPAR<24.61	1 (Ref)						1 (Ref)				1 (Ref)			
NPAR≥24.61	1.69 (1.27-2.25)		<0.001				1.70 (1.28-2.26)		<0.001		1.77 (1.32-2.37)		<0.001	
														

Model 1: Unadjusted. Model 2: Adjusted for age, sex, and race. Model 3: Adjusted for age, sex, race, arrhythmias, hypertension, diabetes, heart failure, thrombolysis, thrombectomy, RBC count, WBC count, platelet count, and SOFA score.

**Table 6:** AUC values and predictive performance of the NPAR.

	AUC	P value	95% CI	Cutoff value	Specificity	Sensitivity	Youden's index
NPAR	0.641	<0.001	0.596-0.686	24.48	0.567	0.684	0.251
Neutrophils	0.610	<0.001	0.564-0.656	80.75	0.644	0.557	0.200
1/Albumin	0.616	<0.001	0.571-0.661	0.351	0.709	0.472	0.180

AUC, area under the curve; CI, confidence interval; NPAR, neutrophil percentage to albumin ratio.
