# Supplementary material for: Neutrophil Percentage-to-Albumin Ratio as a Prognostic Marker for Mortality in Ischemic Stroke Patients
Source: Int J Med Sci. 2025 May 28;22(11):2663–75. doi: 10.7150/ijms.108493 (PMC12163424; doi:10.7150/ijms.108493)
Supplement: Supplementary file 1 — Supplementary figures and tables. [file ijmsv22p2663s1.pdf]

Supplemental Table 1. Missing values of included individuals.

Variable	Missing
**Demographics**	
Age	0
Gender	0
Race	0
Height	0
Weight	0
**Clinical severity**	
GCS	0
SOFA	0
OASIS	0
APS III	0
**Comorbidities**	
Hypertension	0
Diabetes mellitus	0
COPD	0
Heart failure	0
Arrhythmias	0
CAD	0
Cerebral hemorrhage	0
**Laboratory parameters**	
RBC	4(0.57%)
WBC	6(0.85%)
Platelet	4(0.57%)
Hemoglobin	4(0.57%)
Lymphocytes	0
Sodium	0
Glucose	0
Potassium	1(0.14%)
Creatinine	3(0.42%)
Lactate	122(17.28%)
Chloride	0
Bicarbonate	1(0.14%)
Urea	0
**Treatment**	
Thrombolysis	0
Thrombectomy	0

Abbreviation: GCS, Glasgow coma scale; SOFA, sequential organ failure assessment; OASIS, oxford acute severity of illness score; APSIII, acute physiology score III; RBC, red blood cell; WBC, white blood cell;

Supplemental fig. 1. The RCS plots of the NPAR values and mortality rates at 30-day, 1-year, during ICU stays, and during hospitalization in patients with IS before and after PSM.

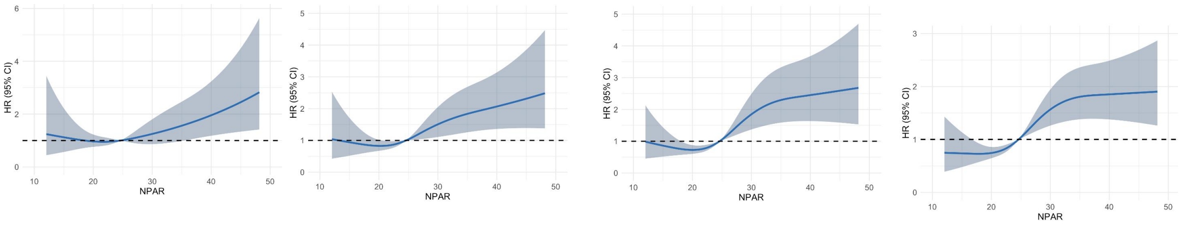

ICU mortality before PSM | In-hospital mortality before PSM | 30d mortality before PSM | 1-year mortality before PSM

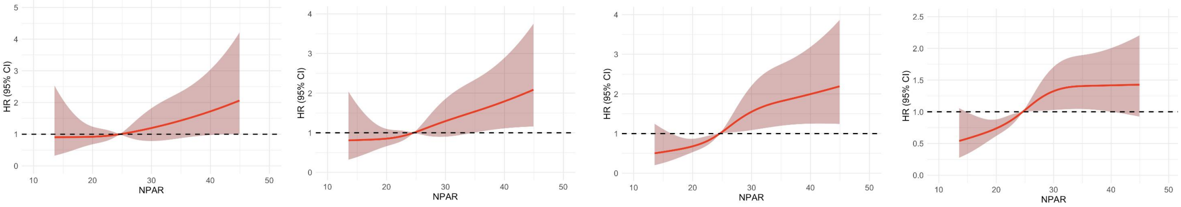

ICU mortality after PSM | In-hospital mortality after PSM | 30d mortality after PSM | 1-year mortality after PSM

Supplemental fig. 2. Subgroup analyses for 30-day, 1-year, and ICU mortality and in-hospital mortality.

Subgroups	ICU-stay all-cause mortality n/total n,(%)	NPAR(>=24.61) HR, 95%CI	P value	P for interaction
Overall	53/353	1.34(0.86-2.11)	0.199	
Hypertension				0.032
Yes	11/52	5.77(1.28-26.06)	0.023	
No	42/301	1.08(0.66-1.74)	0.766	
Diabetes mellitus				0.864
Yes	23/136	1.45(0.72-2.92)	0.297	
No	30/217	1.28(0.71-2.31)	0.408	
CAD				0.277
Yes	10/89	0.95(0.39-2.32)	0.918	
No	43/264	1.55(0.91-2.64)	0.109	
Age				0.927
<70	30/197	1.34(0.75-2.40)	0.317	
>=70	23/156	1.27(0.62-2.62)	0.519	
Gender				0.956
Male	25/185	1.35(0.70-2.62)	0.519	
Female	28/168	1.33(0.72-2.46)	0.375	
Thrombolysis				0.742
Yes	4/19	0.83(0.14-5.00)	0.838	
No	49/334	1.37(0.86-2.18)	0.188	
Thrombectomy				0.912
Yes	2/16	1.68(0.23-12.00)	0.606	
No	51/337	1.34(0.84-2.12)	0.222	

Subgroups	In-hospital all-cause mortality n/total n,(%)	NPAR(>=24.61) HR, 95%CI	P value	P for interaction
Overall	84/353	1.56(1.08-2.26)	0.018	
Hypertension				0.024
Yes	16/52	4.66(1.56-13.95)	0.006	
No	68/301	1.30(0.87-1.93)	0.197	
Diabetes mellitus				0.413
Yes	44/136	1.86(1.08-3.19)	0.024	
No	40/217	1.35(0.81-2.26)	0.254	
CAD				0.348
Yes	19/89	1.23(0.61-2.50)	0.566	
No	65/264	1.72(1.11-2.66)	0.016	
Age				0.901
<70	48/197	1.58(0.98-2.55)	0.063	
>=70	36/156	1.52(0.85-2.72)	0.154	
Gender				0.995
Male	44/185	1.58(0.94-2.64)	0.083	
Female	40/168	1.50(0.88-2.55)	0.135	
Thrombolysis				0.819
Yes	5/19	1.51(0.29-7.96))	0.628	
No	79/334	1.54(1.05-2.25)	0.025	
Thrombectomy				0.800
Yes	3/16	1.41(0.20-10.08)	0.731	
No	81/337	1.54(1.06-2.25)	0.024	

Subgroups	30-day all-cause mortality n/total n,(%)	NPAR(>=24.61) HR, 95%CI	P value	P for interaction
Overall	93/353(26.3%)	2.19(1.53-3.13)	<0.001	
Hypertension				0.036
Yes	19/52(36.5%)	5.58(2.08-14.95)	<0.001	
No	74/301(24.6%)	1.83(1.25-2.69)	0.002	
Diabetes mellitus				0.411
Yes	44/136(32.4%)	2.59(1.50-4.48)	<0.001	
No	49/217(22.6%)	1.92(1.20-3.07)	0.006	
CAD				0.441
Yes	20/89(22.5%)	1.68(0.82-3.44)	0.154	
No	73/264(27.7%)	2.38(1.58-3.59)	<0.001	
Age				0.897
<70	51/197(25.9%)	2.17(1.36-3.46)	0.001	
>=70	42/156(26.9%)	2.22(1.28-3.86)	0.005	
Gender				0.758
Male	44/185(23.8%)	2.05(1.24-3.40)	0.005	
Female	49/168(29.2%)	2.32(1.41-3.85)	0.001	
Thrombolysis				0.401
Yes	4/19(21.1%)	1.07(0.20-5.86)	0.936	
No	89/334(26.6%)	2.26(1.57-3.25)	<0.001	
Thrombectomy				0.977
Yes	4/16(25.0%)	2.06(0.46-9.19)	0.346	
No	89/337(26.4%)	2.20(1.52-3.17)	<0.001	

Subgroups	1-year all-cause mortality n/total n,(%)	NPAR(>=24.61) HR, 95%CI	P value	P for interaction
Overall	172/353(48.7%)	2.19(1.70-2.82)	<0.001	
Hypertension				0.084
Yes	26/52(50.0%)	3.61(1.82-7.17)	<0.001	
No	146/301(48.5%)	1.98(1.51-2.60)	<0.001	
Diabetes mellitus				0.902
Yes	77/136(56.6%)	2.24(1.53-3.27)	<0.001	
No	95/217(43.8%)	2.15(1.53-3.02)	<0.001	
CAD				0.088
Yes	42/89(47.2%)	1.54(0.96-2.46)	0.071	
No	130/264(49.2%)	2.50(1.85-3.38)	<0.001	
Age				0.583
<70	86/197(43.7%)	2.30(1.61-3.28)	<0.001	
>=70	86/156(55.1%)	2.03(1.42-2.90)	<0.001	
Gender				0.761
Male	86/185(46.5%)	2.10(1.48-2.99)	<0.001	
Female	86/168(51.2%)	2.28(1.58-3.28)	<0.001	
Thrombolysis				0.213
Yes	10/19(52.6%)	1.11(0.38-3.24)	0.854	
No	162/334(48.5%)	2.24(1.73-2.91)	<0.001	
Thrombectomy				0.438
Yes	9/16(56.3%)	3.19(1.07-9.57)	0.038	
No	163/337(48.4%)	2.14(165-2.77)	<0.001	
